# The Industrial Future of the South

**Published:** 1891-01

**Authors:** 


### "THE INDUSTRAL FUTURE OF THE SOUTH."

Public Opinion, the eclectic weekly published in Washington and New York, offers a first prize of $50, a second of $30, and a third of $20 for the three best essays on the interesting question: "The industrial Future of the South?" This is a most timely topic, and great interest will be awakened in the competition. The prizes are to be awarded by a committee of three business men of national repute, who will not know the names of the writers until the decision is made. The essays must be limited to 3,000 words, and must be received by December 15th. Full particulars may be had by addressing Public Opinion, Washington, D. C.

